# Cervical myogenic potentials and controlled postural responses elicited by a prototype vestibular implant

**DOI:** 10.1007/s00415-019-09491-x

**Published:** 2019-08-08

**Authors:** Angelica Perez Fornos, Raymond van de Berg, Stéphane Armand, Samuel Cavuscens, Maurizio Ranieri, Céline Crétallaz, Herman Kingma, Jean-Philippe Guyot, Nils Guinand

**Affiliations:** 1 Division of Otorhinolaryngology Head and Neck Surgery, Geneva University Hospitals and University of Geneva, Rue Gabrielle-Perret-Gentil 4, 1205 Geneva, Switzerland; 2 Division of Balance Disorders, Department of ENT, Maastricht University Medical Centre, Maastricht, The Netherlands; 3 Willy Taillard Laboratory of Kinesiology, Geneva University Hospitals and University of Geneva, Geneva, Switzerland; 4 Faculty of Physics, Tomsk State University, Tomsk, Russia

**Keywords:** Vestibular implant, Postural control, Vestibulo-spinal reflex, cVEMPs, Bilateral vestibulopathy, Unterberger/Fukuda test, Stepping test

## Abstract

Gaze stabilization and postural control are two key functions of the vestibular system. In consequence, oscillopsia and chronic imbalance are the two main complaints of patients presenting with a severe bilateral vestibular function loss. The vestibular implant is emerging as a promising treatment for this group of patients whose quality of life is significantly impaired. Although the final aim of the vestibular implant should be to restore vestibular function as a whole, until now the research has focused mainly on the restoration of the vestibulo-ocular reflex to improve gaze stabilization. In this study, we aimed to explore whether the vestibulo-collic and vestibulo-spinal pathways could be activated and controlled with the electrical stimuli provided by our vestibular implant prototype. This was first explored and demonstrated with recordings of electrically elicited cervical vestibular evoked myogenic potentials (ecVEMPs). ecVEMPs with characteristics similar to the classical acoustically elicited cervical vestibular evoked myogenic potentials (cVEMPs) were successfully evoked in five out of the eight tested patients. Amplitudes of the electrically elicited N–P complex varied, ranging from 44 to 120 µV. Mean latencies of the N and P waves were of 9.71(± 1.17) ms and 17.24 ms (± 1.74), respectively. We also evaluated the possibility of generating controlled postural responses using a stepping test. Here, we showed that controlled and consistent whole-body postural responses can be effectively obtained with rapid changes in the “baseline” (constant rate and amplitude) electrical activity delivered by the vestibular implant in two out of the three tested subjects. Furthermore, obtained amplitude of body rotations was significantly correlated with the intensity of stimulation and direction of body rotations correlated with the side of the delivered stimulus (implanted side). Altogether, these data suggest that the vestibular implant could also be used to improve postural control in patients with bilateral vestibulopathy.

## Introduction

Along with visual and somatosensory cues, vestibular sensory input is essential for the sense of balance. The vestibular system works as an inertial motion sensor, which automatically updates the central nervous system with information about head position and motion. Gaze stabilization and postural control are two key features of the vestibular system. The extremely fast vestibulo-ocular reflex is the main contributor to gaze stabilization by generating adequate compensatory eye movements in response to head motion. In addition, the vestibulo-collic reflex also contributes to gaze stabilization by facilitating head stabilization in dynamic conditions [1]. However, in humans, its functional significance remains unknown [2]. Finally, the vestibulo-collic and vestibulo-spinal reflexes significantly contribute to efficient whole-body postural control through the activation of neck, trunk, and limb muscles.

Consistent with the major roles discussed before, in case of a severe bilateral loss of vestibular function, two major and frequent complaints of affected patients are chronic instability and oscillopsia. Both dramatically affect the quality of life. Unfortunately, evidence of an effective, clinically available treatment for this patient population is scarce [3–5]. It has been shown that few specific vestibular rehabilitation protocols can lead to significant functional improvement in this group of patients. However, there is no evidence of long-term benefit [6–8]. Other alternative approaches are currently being explored. Two groups demonstrated that noisy galvanic stimulation applied to the mastoids can have a positive impact on stance and gait. The mechanism underlying the observed improvements is hypothesized to be stochastic resonance. This clearly opens a very interesting line of research; however, the level of evidence of this strategy is still low and its clinical application remains unclear [9, 10]. Another interesting and promising rehabilitation tool is that of the vestibular implant [11]. This is a neuroprosthesis which aims to restore vestibular function by mimicking the physiology of the vestibular system using electrical currents. It comprises motion sensors feeding an external processor which controls an implanted stimulator attached to several electrodes implanted near to the ampullary branches of the vestibular nerve. Special stimulation methods have been developed for a unilateral vestibular implant to restore multidirectional (e.g., up and down, left and right) motion sensations. A “resting” activity of the vestibular nerve is first restored by delivering constant and continuous baseline electrical stimulation. This “resting” neural activity can then be modulated with motion signals to restore vestibular function. Several patients with a severe bilateral vestibular loss have been fitted with vestibular implant prototypes [12]. Our group has demonstrated that it is possible to restore the vestibulo-ocular reflex with a vestibular implant [13, 14]. Moreover, successful functional rehabilitation was demonstrated by showing a significant improvement of dynamic visual abilities while walking [15]. However, although these are encouraging milestones in the development of the vestibular implant, they only represent one aspect of vestibular function: gaze stabilization. Indeed, the other fundamental feature of the vestibular function, postural control, remains poorly assessed, with a single published study showing that electrical stimulation of the semicircular canals can elicit postural responses with a certain degree of specificity for each canal [16].

The present study had two consecutive aims. The first goal was to investigate whether it is possible to activate the vestibulo-collic pathways in patients fitted with a vestibular implant. This was realized by recording electrically elicited cervical vestibular evoked myogenic potentials (ecVEMPs) using a protocol similar to that of the clinical acoustically elicited cervical vestibular evoked myogenic potentials (cVEMP) [17]. Second, in a novel demonstration, we investigated whether it was possible to induce controlled whole-body postural responses while performing a stepping test [18]. Stepping tests such as described by Unterberger or Fukuda are commonly used for the bedside assessment of the vestibular system. However, they are not a specific vestibular test, and their sensitivity and specificity to identify peripheral vestibular lesions in chronic dizzy patients are, in general, poor. There is few available data showing that the sensitivity increased with the severity of the vestibular loss [19]. We did not find consistent data about the sensitivity of the stepping tests in the context of an adequately defined acute unilateral vestibulopathy [20]. We, therefore, made the reasonable hypothesis that an acute loss of the vestibular function mimicked by sudden changes in the artificial “resting” activity restored by the vestibular implant would result in a pathological stepping test with a significant whole-body rotation toward the affected side.

## Materials and methods

Eight patients with a severe bilateral vestibular loss (BVL) received a prototype vestibular implant which consisted of a custom-modified cochlear implant (MED-EL, Innsbruck, Austria) providing one–three extra-cochlear electrodes for vestibular stimulation in monopolar configuration (Table 1). These vestibular electrodes were implanted in the vicinity of the lateral, posterior, and superior ampullary branches of the vestibular nerve (respectively, LAN, PAN, and SAN) using an intralabyrinthine or extralabyrinthine surgical approach [21–23]. Note that some of the electrodes were not tested during these experiments (S3 SAN, S6 PAN, S7 PAN; grayed out in Table 1) since they evoked undesired effects (e.g., facial nerve stimulation) or failed to evoke vestibular percepts in a clinically safe electrical range.

**Table 1:** Main demographic characteristics of the eight patients participating in the study

Patient	Sex	Etiology	Onset	Age at implantation	Year implanted	Implanted side	Vestibular electrodes	Surgical approach
S1	M	Idiopathic	Progressive	68	2007	Left	PAN	EL
S2	M	Congenital / idopathic	Progressive	46	2008	Left	PAN	EL
S3	M	DFNA9	Progressive	67	2012	Left	PAN/LAN/SAN	IL
S4	F	Right mastoidectomy in childhood, left traumatic	Acute(< 1 year)	67	2013	Left	PAN/LAN/SAN	IL
S5	F	DFNA9	Progressive	68	2013	Left	PAN/LAN/SAN	IL
S6	M	DFNA9	Progressive	66	2013	Left	PAN/LAN/SAN	IL
S7	M	DFNA9	Progressive	64	2013	Left	PAN/LAN/SAN	IL
S8	M	Traumatic	Acute(3 years)	53	2015	Right	PAN/LAN/SAN	IL

Note that some vestibular electrodes were not stimulated during the experiments presented here since they evoked undesired effects and they are grayed out in the table

*M* male, *F* female, *PAN* posterior ampullary nerve, *LAN* lateral ampullary nerve, *SAN* superior ampullary nerve, *EL* extralabyrinthine [16, 17], *IL* intralabyrinthine [18]

All patients were recruited at the Division of Otorhinolaryngology and Head and Neck Surgery of the Geneva University Hospitals and at the Division of Balance Disorders at the Maastricht University Medical Center. Strict inclusion criteria were implemented that have been described in detail previously [12, 13].

Only one vestibular electrode was activated for a given experimental trial. All cochlear electrodes were switched off during the entire experimental procedure.

### Electrically elicited cervical vestibular evoked myogenic potentials (ecVEMPs)

The setup for electrical stimulation of vestibular electrodes was composed of a laptop computer running Maestro software (v6, MED-EL, Innsbruck, Austria) that allowed customization of stimulation parameters (current intensity, pulse rate, phase width). The computer communicated this information to the implanted stimulator via a MAX Box system (MED-EL, Innsbruck, Austria) and the cochlear implant system’s antenna.

Seventeen electrodes in the eight patients were tested. The ecVEMPS were recorded with the NeuroAudio system (Neurosoft, Ivanovo, Russian Federation). The recording setup mirrored the standard clinical protocol for cVEMPs. The active electrodes were positioned on the main belly of the sternocleidomastoid muscle (SCM), approximately equidistant from the mastoid process and the sternum. The ground electrode was placed on the superior part of the sternum, and the indifferent electrode on the forehead. The patients were in supine position and were instructed to turn the head contralateral to the stimulation side and then to lift it in order to contract the SCM ipsilateral to the stimulation. Visual feedback of the myogenic activity of the SCM was provided to the patient to ensure a controlled level of muscle contraction during the entire duration of the recording. The stimulus consisted of 100 trials of single cathodic-first, biphasic, charge-balanced pulses delivered at a rate of 5 pulses-per-second to the vestibular nerve by one of the implanted vestibular electrodes. First, a measurement without any electrical stimulation was performed (0 µA) to record baseline noise levels. Then ecVEMPs were measured in two sets of experiments: (1) with varying current amplitude and fixed pulse width (200 µs/phase), where multiple trials were performed with pulses of gradually increasing amplitude, until the upper comfortable level for the patient was reached; and (2) with the current amplitude fixed to the maximum comfortable value reached in (1) but varying pulse widths, starting with a phase width of 200 µs and decreasing it (100 µs/phase, 50 µs/phase) until the ecVEMPS response disappeared. The electromyographic results for each experimental trial were recorded and the average cVEMP wave was then imported to MATLAB R2018b (The Mathworks Inc., Natick, Massachusetts, USA) where the latencies and amplitudes of the positive (P1) and negative (N1) peaks were determined using the function *findpeaks*. When multiple peaks were identified by this function for a given wave, the optimum peak was selected by four experienced clinical observers (authors NG, SC, MR, and APF). Patients were asked to report any percept elicited by the electrical stimulus in all experimental trials.

### Stepping test

The setup for electrical stimulation of the vestibular electrodes was composed of a desktop computer running custom-made research software (MATLAB R2014b, The Mathworks Inc., Natick, Massachusetts, USA) that allowed customization of stimulation parameters (current intensity, pulse rate, and phase width). The computer communicated this information to the implanted stimulator via the manufacturer’s Research Interface Board (RIB II, MED-EL, Innsbruck, Austria) and the cochlear implant system’s antenna.

**Fig. 1:**
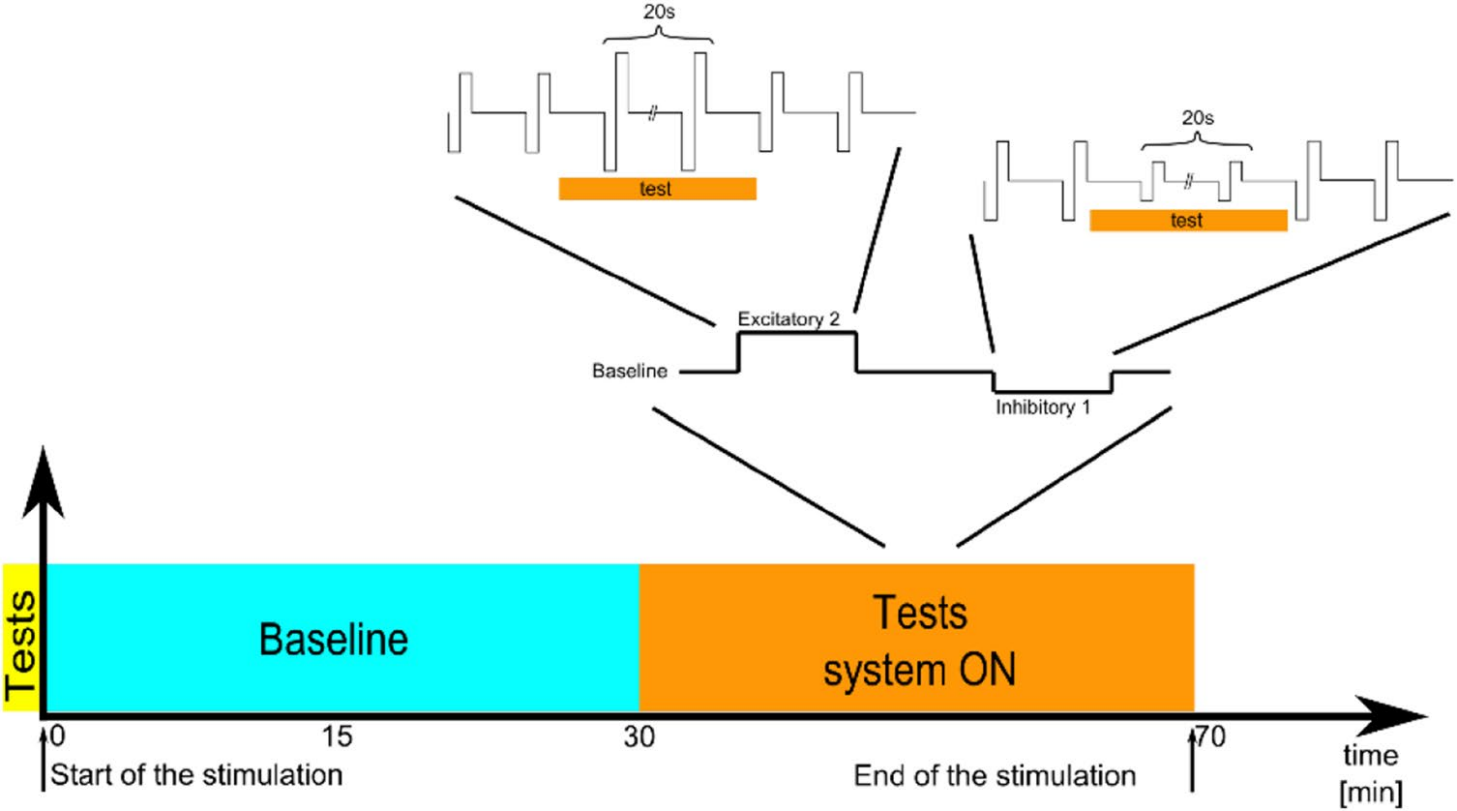
Illustration of the timeline for the experimental protocol of the stepping test. Experimental trials were first performed without any electrical stimulation (system OFF; yellow rectangle) and then in five different conditions of electrical stimulation (system ON; orange rectangle). Note that the system ON conditions included a baseline only condition, two “inhibitory” conditions, and two “excitatory” conditions

Three patients were available for this test (S1, S2, and S4). They were asked to close their eyes, to stretch their arms forward and to perform steps in place. Forty-five to 90° of hip flexion was expected for each step but stepping frequency was not controlled. The duration of each trial was 20 s. Thirty-five reflective markers of 12.5 mm were placed on the skin in relation to bony landmarks according to the Conventional Gait Model (https://doi.org/10.1016/j.gaitpost.2019.04.015). Three-dimensional kinematics were collected using a 12-camera motion capture system (Oqus 7+, Qualisys AB, Gothenburg, Sweden) at 100 Hz. Angular movement (roll, pitch, yaw,) of the head and thorax was computed with the function six degrees of freedom (rigid body) of the Qualisys Track Manager software (Version 2.14, Qualisys AB, Gothenburg, Sweden). Only the yaw angle of the head and thorax (respectively, reported as head rotation angle and thorax rotation angle) was used as outcome parameter. The test was performed without any electrical stimulation (system OFF) and in five different conditions of electrical stimulation (system ON; see Fig. 1). Each condition was tested three times. For each patient, the testing order of the system ON conditions was randomized using a Latin Square and blinded to the all experimenters except the one controlling the electrical stimulation which was not allowed to communicate with others during the experiment. The electrical stimulation profile consisted of charge-balanced, biphasic pulses (200 µs/phase, 400 pulses-per-second) delivered via one vestibular electrode at a time. The five system ON conditions were: (1) baseline stimulation only (described below); (2) and (3) two “inhibitory” conditions with two different current levels (amplitudes) below the baseline current level; (4) and (5) two “excitatory” conditions with two different current levels (amplitude) above the baseline current level. In S1 and S2, the PAN electrode was activated during the experiments; in S4, the SAN electrode was activated.

As previously discussed, before performing the stepping test in the system ON conditions, an artificial “resting” activity of the vestibular nerve in the form of a continuous baseline electrical stimulation had to be restored. This baseline stimulation could then be increased or decreased in amplitude to various levels in the different inhibitory and excitatory conditions. The current amplitude for the baseline stimulation was set at 350 µA for all patients. This value corresponds to the middle of their previously determined dynamic ranges (Table 2). Note that to minimize potential discomfort at the moment of activation, the current was gradually increased (5 s duration ramp) until reaching the determined baseline level. Once the baseline level was reached, the patient was left to adapt to the baseline stimulation for a period of 30 min before starting the system ON experiments [24]. At the end of the system ON experiments, the stimulation was gradually decreased to zero. After each stepping test, patients gave unstructured, subjective feedback of the difficulty of the test. During all stages of this test, two experimenters stood around the tested subject to prevent falls.

**Table 2:** Characteristics of the dynamic range of each of the patients participating in the Unterberger/Fukuda tests (see reference [12] for details on how the dynamic range was determined)

Patient	Electrode	Perception threshold	Upper comfortable level	Dynamic range
S1	PAN	300	400	100
S2	PAN	250	450	200
S4	SAN	300	400	100

All current levels are provided in µA

All subjects gave written informed consent in accordance with the Declaration of Helsinki. Approval of the ethical committees of the Geneva University Hospitals (NAC 11-080) and of the Maastricht University Medical Center (NL36777.068.11/METC 11-2-031) was obtained.

## Results:

### Electrically elicited cervical vestibular evoked myogenic potentials (ecVEMPs)

**Fig. 2:**
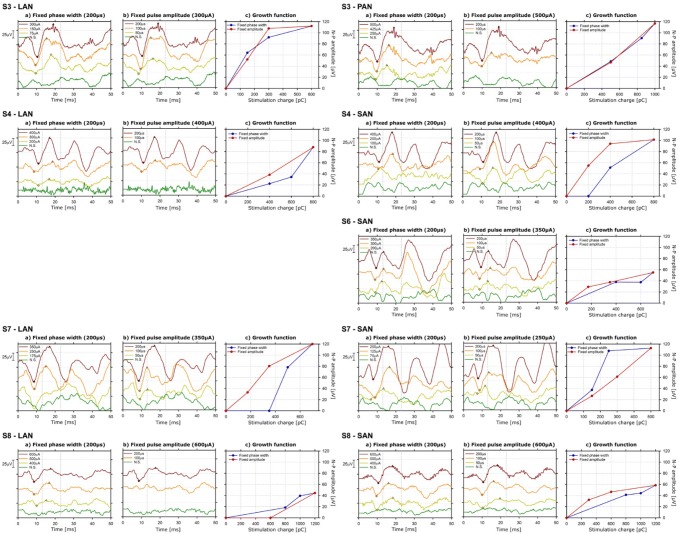
cVEMPs waveforms recorded upon electrical stimulation. Successful recordings were obtained in five patients (9 out of 17 tested electrodes). Each line in the graph corresponds to recordings obtained in one patient, with one vestibular electrode, and one stimulus configuration (phase width and pulse amplitude). The gray dotted lines in the graphs mark the normal latencies of the “classical” acoustical VEMP signals. N.S. stands for no stimulation. Column **a** presents recordings obtained at increasing stimulation currents, and fixed pulse phase (200 µs). Column **b** presents recordings obtained with increasing phase width (i.e., duration), and fixed current amplitude. The detected N and P peaks for each waveform are marked with diamonds and triangles, respectively. Column **c** presents the resulting amplitude/charge relationships (e.g., growth function), both for fixed phase width trials (blue plot) and fixed current amplitude trials (red plot)

ecVEMPs with characteristics similar to the classical cVEMPs could be recorded upon electrical stimulation with 9 out of the 17 tested electrodes in 5 out of 8 patients (see Fig. 2). When ecVEMPs were successfully elicited, the amplitude of the N–P complex increased as the charge contained in the electrical current pulse increased (phase width or current amplitude), as shown in Fig. 2 (columns c). Interestingly, with four out of the nine electrodes having elicited ecVEMPS, varying phase width induced sharper increases in the amplitude of the N–P complex (S4 LAN, S4 SAN, S7 LAN, and S8 SAN). With one electrode, the opposite occurred, current amplitude inducing more marked effects than phase width (S7 SAN). With the remaining four electrodes, the changes induced by both parameters were similar.

The main characteristics of the ecVEMP waves recorded at the maximum charge are presented in Table 3. N–P amplitudes were quite varied across subjects, ranging from 44 µV to 120 µV. Latencies of N and P waves varied with average values of 9.71 ms and 17.24 ms and standard deviations (SD) of 1.17 ms and 1.74 ms for the N and P waves, respectively. None of the patients reported perceiving the stimulus during the experiments.

**Table 3:** Main characteristics of the cVEMPs elicited upon electrical stimulation, at the maximum tested charge (see also Fig. 2)

Subject	Electrode	N–P amplitude [µV]	N latency [ms]	P latency [ms]
S3	LAN	112.11	10.40	19.00
S3	PAN	116.80	10.00	19.00
S4	LAN	87.97	11.00	17.00
S4	SAN	101.51	11.00	18.00
S6	SAN	55.13	9.40	13.40
S7	LAN	120.00	8.60	17.00
S7	SAN	112.70	7.80	16.00
S8	SAN	58.53	10.60	18.20
S8	LAN	44.51	8.60	17.60
	Mean	89.92	9.71	17.24
	SD	29.65	1.17	1.74

### Stepping test

**Fig. 3:**
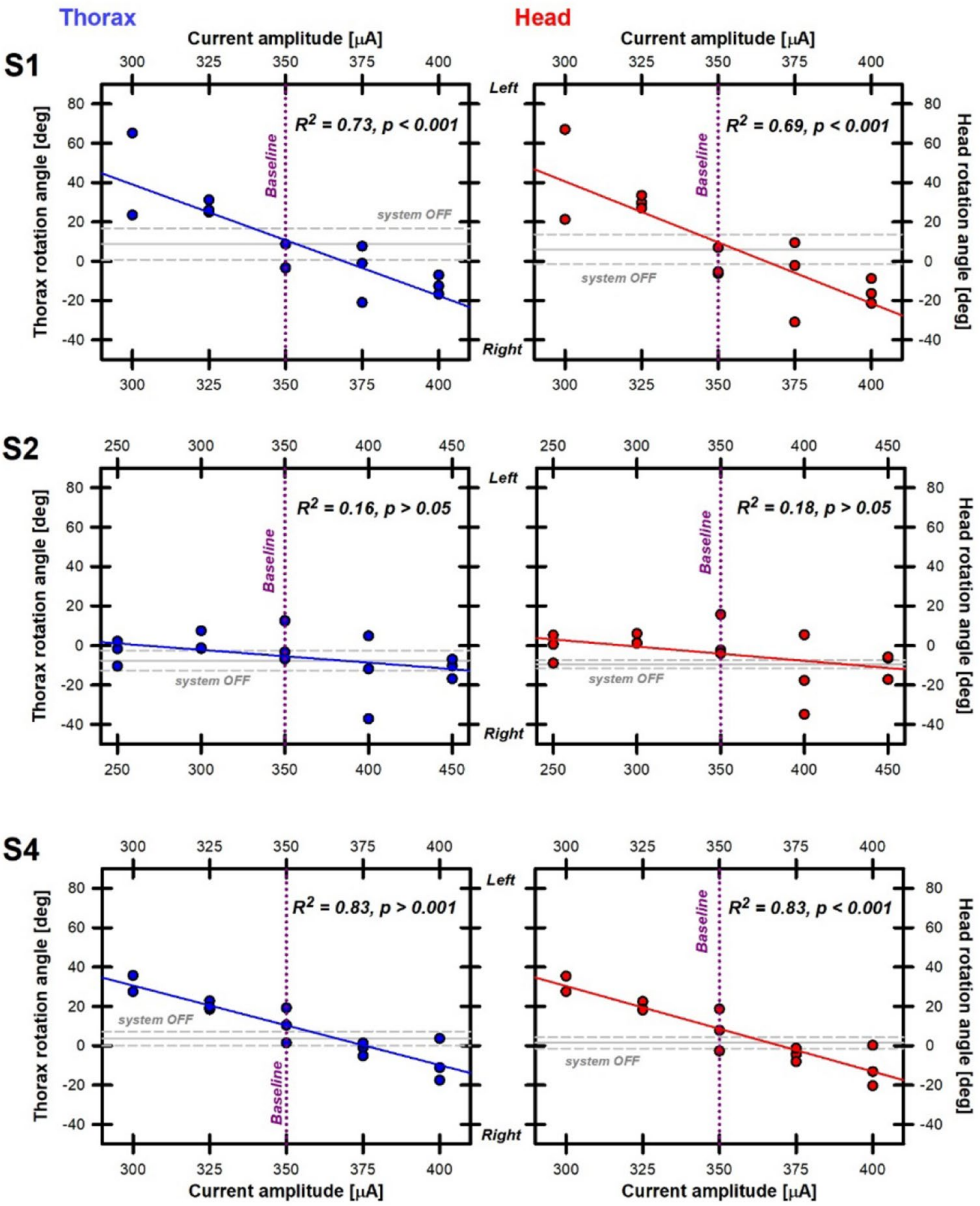
Body rotations versus stimulation current amplitude measured for three implanted subjects (left side). For S1 and S2, the PAN electrode was active, for S4, the SAN electrode. Trunk (blue plots, left column) and head (red plots, right column) were measured during 20 s trials of the stepping test. Three trials were performed per experimental condition (blinded to the subject and the experimenters). “Inhibitory” conditions correspond to stimulation current amplitudes below “baseline” (350 µA, purple dotted lines). “Excitatory” conditions correspond to stimulation current amplitudes above “baseline”. Mean results in the system OFF conditions (without any electrical stimulation) are presented as solid gray lines (± SD, gray dotted lines)

Three patients (S1, S2, and S4) were available for this test. The total angular displacements of the thorax (blue plots) and of the head (red plots) for each patient during each experimental trial, are shown in Fig. 3. Results for thorax and head motion were practically identical (compare head and thorax plots in Fig. 3), demonstrating that the patient’s whole body moved “en bloc”. In the system OFF conditions, the rotations were small, with mean values ranging from − 8° to 9°, consistent with a bilateral vestibular syndrome. No statistically significant difference was observed between results obtained in system OFF condition (gray lines in Fig. 3) and in the “baseline” stimulation conditions (350 µA, purple dotted lines in Fig. 3), demonstrating that patients successfully adapted to the electrical stimulation (see Table 4). We observed strong and significant negative correlations between current intensity and thorax/head rotation in S1 and S4 (Pearson product-moment correlations: S1 thorax *R* = 0.85, *R*^2^ = 0.73, *p* < 0.0001; S1 head *R* = 0.83, *R*^2^ = 0.69, *p* < 0.001; S4 thorax *R* = 0.92, *R*^2^ = 0.83, *p* < 0.0001; S4 head *R* = 0.92, *R*^2^ = 0.83, *p* < 0.0001). “Inhibitory” conditions resulted in head and thorax rotations toward the implanted side (left) reaching rotation values above 30°–40° while “excitatory” conditions resulted in rotations toward the non-implanted side (right) below − 15°. Note that the rotations obtained for S2 were similar in all conditions and the correlations were non-significant (Pearson product-moment correlations: thorax *R* = 0.40, *R*^2^ = 0.16, *p* = 0.13; head *R* = 0.43, *R*^2^ = 0.18, *p* = 0.10). Linear body motion (distance and direction) was varied and uncorrelated to electrical stimulation for all patients in all experimental trials (data not shown).

**Table 4:** Statistical comparison (paired* t* test) between system OFF and “baseline” stimulation results

Patient	Thorax			Head		
	System OFF	Baseline		System OFF	Baseline	
S1	8.74 ± 7.99	0.62 ± 6.99	*t* = 1.106, *p* (two-tailed) = 0.384	6.11 ± 7.57	− 1.57 ± 7.39	*t* = 0.997, *p* (two-tailed) = 0.424
S2	− 7.56 ± 5.07	0.84 ± 10.27	*t* = − 1.022, *p* (two-tailed) = 0.414	− 9.50 ± 2.09	3.13 ± 10.85	*t* = − 1.694, *p* (two-tailed) = 0.232
S4	3.59 ± 3.60	5.95 ± 6.39	*t* = − 1.200, *p* (two-tailed) = 0.442	1.48 ± 2.85	2.55 ± 7.47	*t* = − 0.328, *p* (two-tailed) = 0.798

Results in each condition are presented as mean ± standard deviation

## Discussion

The goal of this study was to investigate whether the vestibulo-collic and vestibulo-spinal reflexes could be elicited and modulated in human patients implanted with vestibular implant prototypes. The results confirm that direct application of electrical currents to the ampullary branches of the vestibular nerve via chronically implanted electrodes is an effective way of activating the vestibulo-collic pathway and of inducing controlled postural responses. We hypothesized that the latter results from the activation of the vestibulo-spinal pathways as also demonstrated in animal studies [1]. Furthermore, increasing the energy in the electrical stimulus also increased the response, allowing the modulation of the activity with the corresponding postural responses.

All patients participating in the study could complete the experiments without complications. An ecVEMP response could be observed in five out of the eight tested patients. Nine out of the 17 tested electrodes were responsive in this patient group. Although the shape of the ecVEMP was similar to the classical acoustically elicited cVEMP, the latencies of the former were shorter, similar to previous observations in studies comparing galvanic to acoustic stimulation [25]. We hypothesized that bypassing the mechanoelectrical transduction explains this difference. In the stepping test, two out of three patients showed significant whole-body postural responses, S1 and S4. These “en bloc” whole-body postural rotations followed our initial hypothesis: a significant thorax rotation with a direction away from the stimulated side was obtained when an acute unilateral vestibulopathy was mimicked by a sudden decrease in the current amplitude delivered by the vestibular implant. The opposite was true when the current amplitude was suddenly increased, mimicking an acute unilateral “irritative” process. The obtained thorax rotation amplitude was correlated to the magnitude of the sudden current amplitude change. In S2, no significant differences were found between the different conditions, although the trend in the results was in the same direction as for the other subjects. It is to be noted that in S4 the active electrode was the SAN electrode, while in S1 and S2, the stimuli were delivered through the PAN electrode. Unfortunately, our small sample size does not allow us to draw any conclusions with respect to the relationship of the location of the active electrodes (PAN or SAN) and the response. If predominantly of ampullary origin, it could have been speculated that the strongest responses would have been obtained with an active electrode in contact with the LAN, as the resulting thorax rotations were in the yaw plane. This requires further investigation as no LAN electrode was tested in this experiment.

Previously, Philipps and al. demonstrated postural responses upon electrical stimulation with a vestibular implant in four patients [16]. They showed that in a same patient, stimulation of the anterior canal resulted in a sway in opposite direction to the sway obtained when stimulating the posterior canals. Stimulation of the lateral canal produced a medio-lateral sway directed toward the contralateral ear in addition to the antero-posterior component. However, these results are not directly comparable with ours since their setting was significantly different. First, the patient profile was different, as they included patients with severe unilateral Menière Disease with heterogeneous post-operative residual vestibular function (two unilateral vestibular impairments and two bilateral vestibular impairments). In contrast, the patients included in our study suffered with severe bilateral vestibular loss, and the results of the stepping test in the system OFF condition were consistent with this pathology. Second, in the study by Phillips et al. [16], the stimulation paradigm consisted of acute trains of stimulation lasting 2 s while patients were standing still on a platform. In our study, patients first adapted to a baseline electrical activity that was subsequently modulated in inhibitory and excitatory conditions. Altogether, these important differences significantly restrict meaningful comparisons with the results obtained in the current study. The stimuli used to elicit the ecVEMP were not perceived by any of the tested subjects. This can indicate that with this particular stimulation profile, the vestibulo-collic pathway shows a lower threshold than the pathways mediating vestibular perception. In previous experiments performed with a different stimulation profile, we observed lower thresholds for perception than for the vestibulo-ocular reflex (unpublished data). This observation strengthens the hypothesis that the different vestibular pathways have different activation profiles. During the stepping test, all three patients reported that the test was challenging for them due to imbalance and a certain degree of fear to fall. This impression was increased in both the low- and high-current amplitude conditions. Patients did not report other vestibular or non-vestibular (i.e., auditory, pain, facial twitch…) symptoms during the tests.

In humans, there is a good evidence for a saccular origin of the acoustically elicited cVEMPS, while the extent to which the canals contribute to vestibulo-collic/spinal reflexes remains poorly known. Since the main stimulation targets with our vestibular implant are the afferents of the semicircular canals, one can hypothesize that the reported responses originate from these structures. This, of course, necessitates that the stimulating electrodes are positioned as close as possible to the neural tissue of each ampulla. This achievement remains to be a partly unsolved challenge since there still remains a significant variability of each electrode position with respect to the targeted neural structures. Nevertheless, based on CT scans and characteristics of the vestibulo-ocular responses (both not shown in this study), we are confident that the ampullary branches of the vestibular nerve were the primary targets of the delivered electrical stimulation [12]. This supports the hypothesis that canals can elicit and modulate vestibulo-collic/spinal reflexes and is in line with results in animal research showing that neck motoneurons responded to stimulation of any of the six canals [26]. A potential ampullary origin of vestibulo-spinal responses has also been reported in the first pioneering studies of Cohen and Suzuki [27], where clear and reproducible postural responses could be observed upon electrical stimulation of semicircular canal afferents in animals [27]. An alternative hypothesis is that in our experiments, the activation of the vestibulo-collic and the vestibulo-spinal pathways could be due to unintended current spread from the semicircular canals to otolithic structures. This is difficult to confirm and/or quantify experimentally. In conclusion, our results, taken together with previous animal research, indicate that the ampullary origin of the vestibulo-collic and vestibulo spinal responses observed in this study is probable but an otolithic contribution through current spread cannot be entirely ruled out.

In summary, the results presented here provide an additional piece of evidence of the potential of the vestibular implant to rehabilitate patients with a severe bilateral loss. After all, the possibility of restoring controlled postural responses addresses a highly relevant issue in this population. In addition, it has been suggested that the vestibulo-collic reflex might also be highly complementary to the vestibulo-ocular reflex for effective gaze stabilization abilities. This also appeared to be the case in our own previous studies where we observed significant improvements in dynamic visual abilities that could not be fully explained by the artificially evoked vestibulo-ocular reflexes [15]. Future work must, therefore, carefully investigate the following two aspects: (1) how and to what extent each of the different vestibular pathways can be selectively activated, and (2) how each evoked vestibular response contributes to the restoration of everyday activities. On one hand, this will be essential to optimize processing and stimulation strategies to fully exploit the rehabilitation potential of the vestibular implant. On the other hand, this unprecedented new data should contribute to improve our understanding of the interaction of the vestibular end organs and pathways in the complex, multisensory sense of balance.
